# Prediction of VMAT gamma passing rates using 3D CNNs based on leaf position analysis and gradient class activation mapping for plan complexity evaluation

**DOI:** 10.1002/mp.70468

**Published:** 2026-05-05

**Authors:** Johannes Berchtold, Sara Vockner, Ivan Messner, Markus Stana, Falk Röder, Frank Wolf, Christoph Gaisberger

**Affiliations:** ^1^ Department of Radiation Therapy and Radiation Oncology Paracelsus Medical University Salzburg Austria; ^2^ Institute of Research and Development of Advanced Radiation Technologies (radART) Paracelsus Medical University Salzburg Austria

**Keywords:** 3D CNN, Grad‐CAM, VMAT QA

## Abstract

**Background:**

Volumetric Modulated Arc Therapy (VMAT) is a highly conformal radiotherapy technique that enables precise tumor irradiation while sparing surrounding healthy tissue. However, the high technical demands this technique places on Linear Accelerators (LinAc) necessitate reliable quality assurance (QA) tools. The Gamma Passing Rate (GPR), commonly used to compare planned and delivered dose distributions, requires extensive measurement resources. Many existing predictive metrics, such as the popular Modulation Complexity Score (MCS), are independent of the beam model, limiting their accuracy. Consequently, identifying appropriate metrics and their individual thresholds can be challenging.

**Purpose:**

This study aims to predict the GPR of VMAT arcs using a three‐dimensional convolutional neural network (3D CNN). Gradient‐weighted Class Activation Mapping (Grad‐CAM) is applied to improve interpretability, identify weak segments, and potentially reveal beam model limitations.

**Methods:**

A 3D CNN was trained on 140 6 MV VMAT arcs, with 30 arcs each used for validation and testing. All plans were delivered by an Elekta Harmony Pro LinAc with 4° control point (CP) spacing. Input data included discretized beam's eye view (BEV) representations and segment‐specific monitor unit (MU) values. GPR evaluation was performed using a Delta4+ phantom with a 1%/ 2 mm criterion. Data augmentation enhanced training diversity. Grad‐CAM was used to visualize influential plan regions.

**Results:**

After 36 epochs, the model achieved a mean absolute error (MAE) of 2.0%(test set) and 1.3%(training set). With cropped input, the best MAEs were 2.1%(test) and 1.5%(training). Grad‐CAM analysis indicated that dynamic delivery aspects had more influence on prediction accuracy than static features like field shape.

**Conclusions:**

This study highlights the potential of deep learning for automated GPR prediction, offering a more efficient QA workflow. Especially in time‐critical settings like online adaptive radiotherapy, where traditional measurement‐based QA is often impractical, this model provides a scalable solution to ensure treatment safety. The use of Grad‐CAM enables insight into beam model and LinAc performance, allowing refinement of treatment planning and improved QA precision in clinical practice.

## 1 INTRODUCTION

Volumetric Modulated Arc Therapy (VMAT) is a technique that can push the technical capacities of a linear accelerator (LinAc) and its multileaf collimator (MLC) to their limits.^1^ As VMAT involves complex treatment planning, it is essential to verify the accuracy of the delivered dose as recommended by the AAPM Task Group No. 218.^2^ Gamma analysis is the gold standard for dose comparison, it is commonly used to compare planned and delivered dose distributions in order to assess the agreement and ensure the quality of radiation therapy for each individual patient.^3^, ^4^ Although secondary dose calculation algorithms can also be employed for plan verification, they cannot fully capture performance characteristics of the LinAc itself.^5^ In practice, deviations in treatment delivery arise not only from the beam model but also from machine‐specific factors such as MLC leaf speed, which has a well‐documented impact on dose accuracy.^5^, ^6^

Conventional methods for performing gamma analysis require manual measurements, which are time consuming. To reduce the load of patient specific quality assurance (PSQA) on the machine, researchers have introduced several different metrics, such as the Modulation Complexity Score (MCS), the Closed Leaf Score (CLS), Small Aperture Score (SAS) and many more attempting to access treatment plan quality without measuring the corresponding dose distribution.^3^, ^7^, ^8^

However, those metrics are calculated independent of the underlying beam model, where small inaccuracies can have a significant effect on VMAT QA results.^9^ It has also been shown, that the different plans differ in their optimal beam model parameters.^10^ This is not necessarily reproduced by the metrics mentioned above. For example, if a beam model more accurately represents small fields than larger ones, the SAS becomes an unreliable parameter, since it focuses more on small segments. This becomes even more evident when considering that two geometrically identical treatment plans, calculated with different beam models, will have matching geometrical metrics but will differ significantly in their Gamma Passing Rate (GPR).

Several machine learning models have already been developed to predict the GPR based on the above mentioned plan complexity parameters within reasonable accuracy.^11^ Valdes et al. pioneered prediction based PSQA, employing 78 aperture‐based complexity metrics and a Poisson regression with Lasso regularization to predict the GPR of IMRT plans.^12^ Wall et al. extended this approach to 241 parameters derived from treatment plan data—segment‐specific values such as gantry angle, monitor units (MU) and leaf positions.^13^

More recently, also Convolutional Neural Networks (CNN) have been applied to GPR prediction, drawing on a wide range of inputs, including complexity metrics, dose distributions, MLC parameters, or a combination of those.^11^, ^14^, ^15^, ^16^, ^17^, ^18^

However, we want to show with this work, that only the core elements of a treatment plan, specifically the basic information that the LinAc receives, suffice for GPR prediction. Those core elements—the segment‐specific leaf positions combined with the corresponding segment MU—can be visualized in the beam's eye view (BEV), with consecutive segments shown as image series with each image corresponding to a specific gantry angle. We aim to predict GPR using a 3D CNN on the BEV images, as this architecture is well suited for extracting features from image sequences.^19^

Rather than replacing conventional PSQA, this prediction approach is intended as complementary tool—for example, as addendum to routine machine QA—to help identify cases that may benefit from additional measurements. If the predicted GPR falls below a specific threshold, further steps such as PSQA should be initiated.

While this method has the potential to streamline QA workflows by reducing unnecessary measurements, it cannot eliminate the risk of creating plans failing validation. The root cause of such failures may remain unknown, especially in cases where the beam model accuracy is not in doubt.

Several publications have discussed instances where a beam model was tested and well approved for clinical use, however, as the planning system was applied to more complex treatment sites, the plans started to fail the institution's measurement‐based plan PSQA tests.^20^ Possible reasons are algorithm weaknesses or unavoidable measurement uncertainties in the commissioning phase.^21^, ^22^

To address this challenge, we integrate Gradient‐weighted Class Activation Mapping (Grad‐CAM) into our model.^23^ Grad‐CAM is a technique for explainable AI which provides visual explanations for the model's predictions by highlighting the most important regions in the input that contributed to a specific decision. This is achieved by computing the gradients of the target class with respect to the feature maps of a chosen convolutional layer and the weighting of these feature maps accordingly to generate a heatmap. This heatmap is overlaid on the original input, allowing for an intuitive interpretation of the model's focus areas.

This technique allows us to gain deeper insights into the underlying causes of poor performance in the gamma analysis, highlighting specific areas of the treatment plan that may be contributing to suboptimal validation results. By applying this method, we can identify and address potential weaknesses in the plan, ultimately leading to more accurate and reliable treatment planning strategies and an overall better understanding of the beam model in use. Furthermore, with the integration of Grad‐CAM, visual input now plays a crucial role. This provides a visual representation of the model's focus areas in BEV, enhancing the interpretability and transparency of the prediction process. This is in contrast to complexity metric‐based models, which do not allow interpretability on such a scale.

## 2 METHODS

The dataset used in this study consisted of 113 6 MV VMAT plans generated with the treatment planning software (TPS) RayStation (RS) (RaySearch Laboratories, Stockholm, Sweden). Plans are derived from clinical data for a variety of patients and entities, such as breast cancer, head‐and‐neck cancer, rectal cancer, lung cancer, spinal metastases, and extremity tumors. The CNN is believed to be generalized to an extent that treatment site selection is deemed unnecessary. Treatment plans were chosen continuously in the order they were created during daily clinical routine. If a single arc treatment plan was not sufficient for achieving adequate plan quality, a dual arc plan was created, using the “create dual arc” feature.

In RS, dual arc treatment plans are displayed as two separate individual arcs. However, two arcs within the same plan are not independent in the strict sense, but they are optimized by the TPS algorithm to complement each other when using the “create dual arc” feature. These differences are reflected in the individual arcs’ GPR. For model training, we treated each of these individual arcs separately, splitting dual arc plans into their corresponding single arcs. This approach resulted in expanding the dataset to a total of original 200 single arcs, which were then split random into training, validation and test sets. The training and validation sets were split on arc level, the test set was strictly split on patient level to prevent data leakage.

A 4° control point (CP) spacing was used for treatment planning, resulting in 90 segments for a complete 360° VMAT treatment arc, with each segment corresponding to a specific gantry angle. For each segment, the 160 leaf positions and the corresponding MU were extracted. To address missing arc angles, padding was applied by inserting empty (zero) segments to fill these gaps and maintain a uniform angular distribution. The data was exported from the TPS as numpy files.

Over a period of three weeks, eleven dose measurement sessions were conducted to measure the GPR of the plans used for model training. To evaluate the accuracy of the GPR measurements, six specific treatment plans were analyzed across those measurement sessions. The measurements were obtained using an IMRT phantom (Delta4+, ScandiDos, Sweden) with a 2 mm dose grid for phantom dose calculation. Dose delivery was carried out with an Elekta Harmony Pro LinAc, equipped with an Agility Head (160‐leaf collimator, 80 leaves/leaf‐bank, leaf width @ isocenter = 5 mm) and a maximum field size of 400 mm × 400 mm. The Delta4+ phantom was positioned using standardized rectangular irradiation fields, with adjustments made according to the optimal phantom position. The GPR pass criteria were set to 1%/ 2 mm with a global normalization and a cut‐off at 10%of the maximum dose. This was chosen instead of the TG‐218 recommended 3%/ 2 mm, because that threshold would result in overly favorable GPR values, limiting the model's ability to train effectively due to a lack of GPR variability.

Python was used for further data preparation, which consisted of the following components:

**Data representation for treatment arcs**: For each segment of a treatment arc, an 80 × 400 matrix was created, with all entries initially set to zero. This results in a 90 × 80 × 400 tensor for a complete treatment arc. The first dimension represents the temporal aspect, with each index corresponding to a specific gantry angle, the second‐dimension indexes the 80 leaf pairs (the y‐axis of the BEV) and the third dimension represents the leaf positions in the x‐direction.

This discretization of the BEV has a resolution of 5 mm (leaf width) × 1 mm, where each row corresponds to a leaf pair.

**Leaf Position Handling**: The leaf positions were rounded to the nearest integer and mapped to indices in the x‐direction. The values between two indices of a leaf pair were set to the segment‐specific MU value, provided the leaf pair was not hidden behind the aperture.

**FIGURE 1:**
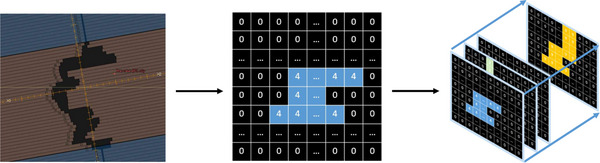
Translating the BEV into numpy files. A segment is translated into a matrix and following segments are lined up to represent the VMAT arc.

For example, for a segment with 4 MU, the corresponding 80 × 400 matrix would be populated as shown in Figure 1.

**Optional modification**: The measurement volume of the Delta4+ is only 20 cm × 20 cm. Crop each segment matrix to size 40 × 200, corresponding to a 200 mm × 200 mm BEV. Cropped input is later compared to input using the full BEV.

**Data augmentation**: Solely symmetry considerations were used to quadruple the training dataset: (1) flip each BEV about the Y‐Axis. (2) flip the BEV around the X‐Axis and (3) rotate the BEV by 180° about the Z‐Axis, which corresponds to an X‐flip combined with a Y‐flip. The augmentation function was implemented exclusively within the training process to prevent data leakage. To estimate the uncertainty this method adds to the input data, ten arcs where randomly chosen and measured together with their augmented siblings.

The model utilized in this study is a 3D CNN built using the deep learning library Keras and the TensorFlow framework as backend.

**FIGURE 2:**
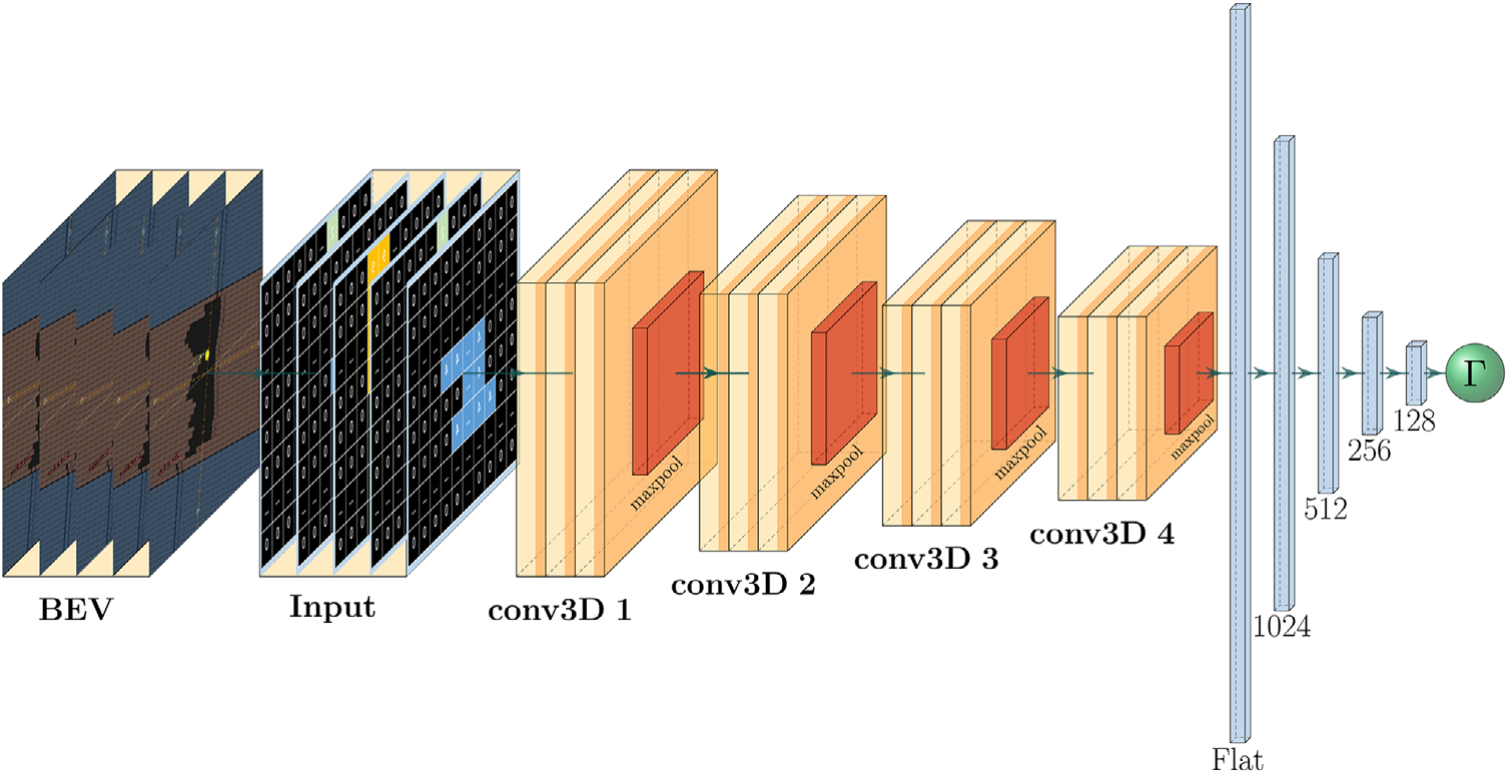
The model consisting of four 3D convolutional layers and four dense layers using ReLu activation.

The architecture as shown in Figure 2 includes convolutional layers, that is, four 3D convolutional layers with 32, 64, 128, and 256 kernels, a kernel size of (3, 4, and 4) and ReLU activation. Each layer is followed by a 3D max pooling layer with a pool size of (2, 2, and 2). Furthermore, it includes fully connected layers, where after flattening the output, four dense layers with 1024, 512, 256, and 128 units, using ReLU activation, lead to a final output layer with a single neuron—the GPR.

The model was compiled using the Adam optimizer with Mean Squared Error (MSE) loss and Mean Absolute Error (MAE) as the evaluation metric and was trained with a training set/validation set/test set ratio of 0.7/0.15/0.15, batch size 4 and 75 epochs. Regularization was used to prevent overfitting. Random splits were employed.

The complex architecture of CNNs prevents a holistic understanding of the decision‐making process in detail. However, there are several possibilities, such as Grad‐CAM, to gain insight in the networks function.^24^ In our study, Grad‐CAM is not only used for debugging purposes, but also to enhance interpretability, i.e. to help us understand which features of a treatment plan influence the prediction process. In our case, we are especially interested in features leading to a low GPR.

The model with the lowest validation MAE was used for Grad‐CAM.

To validate the Grad‐CAM findings in at least one arc, we measured all segments of the selected VMAT arc (partially shown in line III Figure 5) as static fields. In addition, we created a modified VMAT plan consisting of the four consecutive segments shown in Figure 5, line III, periodically repeated over a full arc, which was also measured. This was implemented to test the dynamics of these consecutive segments.

## 3 RESULTS

The same six arcs measured before each measurement session, resulted in an average GPR of 94.2%. These arcs showed an average absolute variation of 2.1% and a maximum deviation of 4.0%.

The ten plans measured along with their augmented counterparts, showed an average deviation of 2.9%, and a maximum deviation of 5.2%.

**FIGURE 3:**
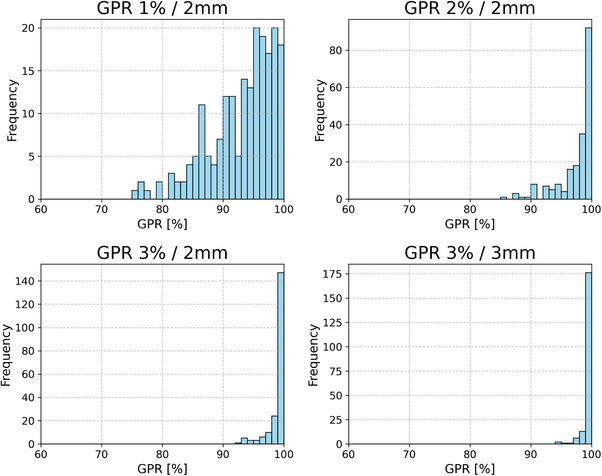
Histograms of GPR for different criteria. In the 3%/ 2 mm criterion, 35 arcs have a GPR < 85%, mean GPR is 93.0%, in the 3%/ 2 mm criterion, 15 arcs have a GPR < 95% and the mean GPR is 99.1%.

For the 200 measured treatment arcs, mean GPR (1%/ 2 mm criterion) was 93.0%, with a minimum of 75.6% and a maximum of 100% and a standard deviation of 6.0%. Median was 94.7%. Mean absolute deviation (MAD) between mean GPR and the actual GPR is 4.7%, between median and actual GPR is 4.6%. Figure 3 shows the GPR distributions for different passing rate criteria. All arcs with a GPR < 95% with the 3%/ 2 mm criterion had a GPR < 85% with the 1%/ 2 mm criterion.

### 3.1 VMAT GPR Prediction

The best MAE for the validation set was achieved using the complete BEV as input after 36 epochs, with a training MAE of 1.3%, a validation MAE of 2.2% and a test MAE of 2.0%.

With cropped training data, best training MAE and validation MAE converged at 1.5% and 2.4%, respectively. The test MAE was 2.1%.

We analyzed the training trajectories for both the full BEV and the cropped BEV, comparing the best MAE of the validation set for each. The cropped BEV performed the worst, with the poorest model not achieving a validation MAE lower than 3.3% over the 75 epochs. There was only one other model (also with cropped BEV) that did not achieve a MAE below 3.0% for the validation set.

**FIGURE 4:**
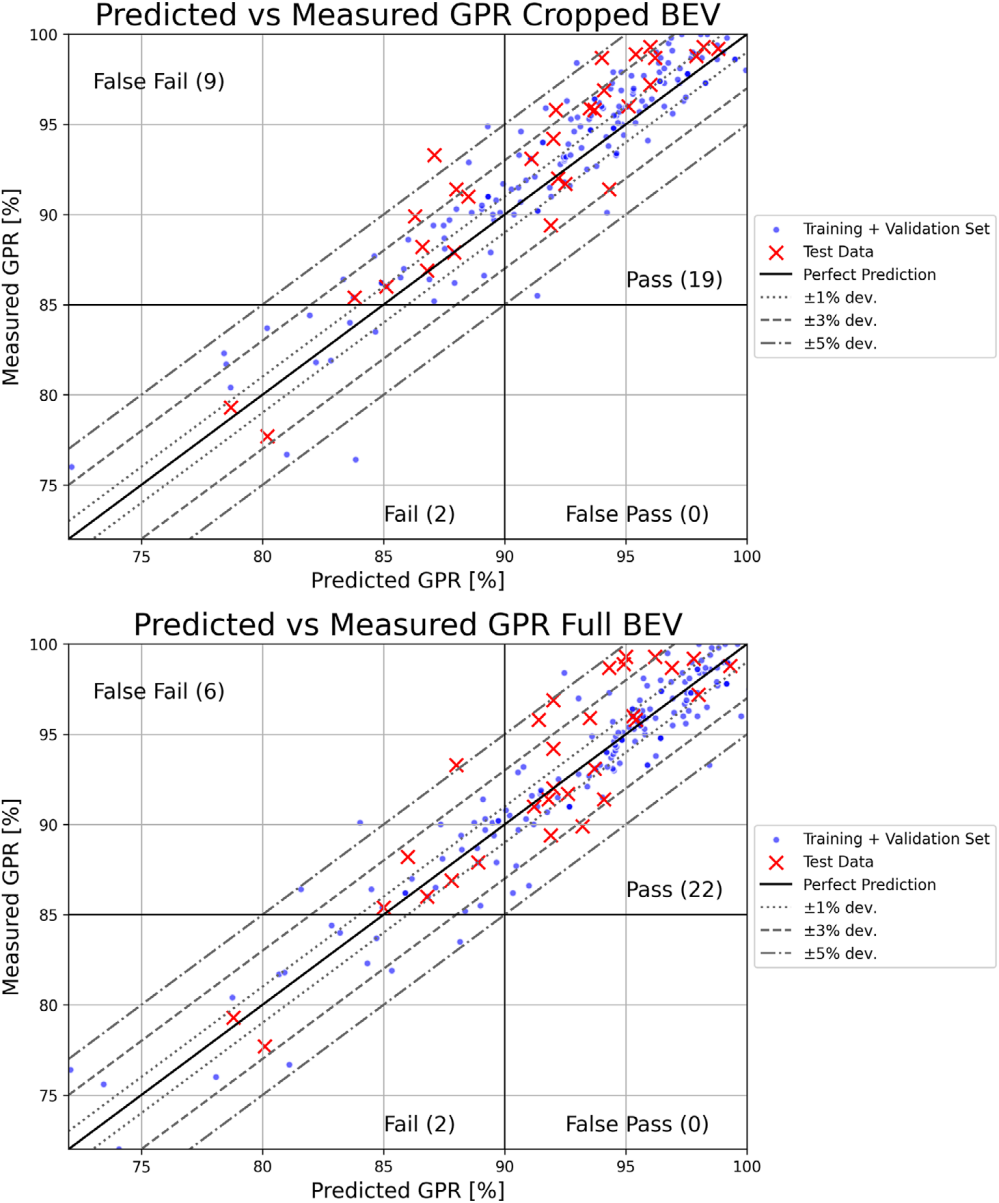
Scatter plots comparing the measured and predicted GPR for the cropped BEV input (top) and full BEV input (bottom). The full BEV input demonstrates higher predictive accuracy, with 43% of test set arcs predicted within ± 1% deviation, compared to 30% for the cropped input. The maximum deviation observed was 6.1% for the full BEV and 7.4% for the cropped input.

The slight superiority of the full BEV can also be seen by examining the predictive accuracy of the individual plans, shown in Figure 4. In both cases, applying a prediction threshold of 90% GPR results in zero false‐pass cases, meaning that no arcs with a measured GPR below 85% go undetected. In our case, this would mean that setting this threshold finds all arcs with a GPR < 95% with the 3%/ 2 mm criterion.

### 3.2 Visual representation using Grad‐CAM

**FIGURE 5:**
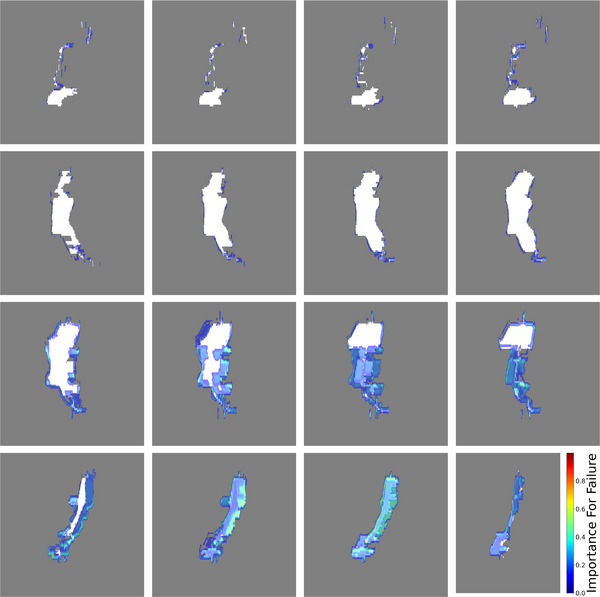
Grad‐CAM heatmap overlay on BEV. The BEV is shown as white shape, where the importance for GPR‐failure is overlayed as heatmap. Each row shows four consecutive segments all of which belong to the same treatment arc, with a GPR of 80.4%. First and second rows show four consecutive segments with almost zero dynamics, with low importance for failure, third and fourth rows show two different parts with lots of leaf‐movement, with a higher importance for failure.

The generated arc‐specific Grad‐CAM heatmaps highlighted the regions within the segments of the arc that had the most significant impact on the model's predictions. These importance heatmaps allowed us to pinpoint specific areas within the beam delivery process that were responsible for deviations in dosimetry accuracy, as shown in Figure 5.

Segment sequences with high leaf dynamics, that is, segment sequences where field shapes change drastically between consecutive CP exhibit a higher importance for failure than static segments, independent of the field shape. Consecutive segments, in which one half of the jaw‐defined aperture is dynamically blocked by the MLC leaves from segment to segment while the opposing half remains open (see row III in Figure 5), are prone to result in a low GPR. Since this is not a trivial task to test, we only tested this specific sequence consisting of 4 consecutive segments, which were repeated through an arc with 90 CP. In addition, the individual segments were measured as static fields. The static fields (100 MU each) performed well in the gamma analysis, whereas the periodic arc failed, showing a GPR of 73.4%.

## 4 DISCUSSION

Artificial Intelligence is increasingly used in various areas of radiotherapy.^15^ Naturally, several prediction tools have already been implemented, particularly in the area of QA. Tomori et al. used a CNN for GPR prediction of prostate plans, with sagittal planar dose distribution from a QA phantom, volume of the PTV and rectum and the overlapping region between them and the MU values for each irradiation field as input.^17^ Tozuka et al. developed another effective approach in which the GPR of VMAT plans was predicted using MLC leaf positions, with dose distribution data from the patient's CT scan serving as model input.^18^ Hao et al. used a neural architecture search to predict successfully the GPR of IMRT plans, with fluence maps as input.^14^

Nonetheless, we chose to use only the essential CP‐specific attributes—leaf positions, gantry angle and MU—as model inputs. This minimal set of information corresponds to what a treatment plan requires and what the LinAc receives for further processing. Furthermore, we intended to utilize the trained model to gather feedback, which is why we opted to implement Grad‐CAM. As a result, the feedback can be displayed directly onto the graphical input, enabling visual interpretation of PSQA failure points. Based on this feedback, we can adjust the beam model or RS settings.

In this discussion section, we will explore the implications of our findings in relation to the effectiveness of 3D CNNs for predicting GPR in VMAT treatment arcs. By examining the insights gained from Grad‐CAM analysis, we aim to deepen our understanding of the underlying factors influencing model predictions and discuss potential avenues for future research to enhance model performance and clinical applicability.

### 4.1 VMAT GPR Prediction

The results of this study demonstrate that 3D CNNs are effective at predicting GPR for VMAT treatment arcs based on CP‐specific leaf position data and MU. The model achieved a test MAE of 2.0%, which is within the measurement uncertainty range of 2.1%, indicating that the model's predictions are reliable and consistent with the inherent uncertainty in GPR measurements.

To estimate the range of this uncertainty, six selected treatment arcs were measured and the GPR was evaluated prior to each of the 11 measurement sessions. With an average deviation of 2.9% and a maximum deviation of 5.2%, these results demonstrate the sensitivity of the GPR using the 1%/ 2 mm criterion. However, such an aggressive criterion was mandatory in our case, as the training data set was still unbalanced—especially compared to a less aggressive criterion.

The unbalanced training dataset underscores the importance of intentionally generating plans, which have a low GPR, to ensure that plans with low GPR are effectively identified. The need for more plans with a low GPR in the training set can also be seen in Figure 3 as 16 arcs had a measured GPR < 85%.

While we observed that augmented arcs exhibit slight differences in GPR measurements, these differences remained within the measurement uncertainty range, allowing us to include these arcs in the model training. On a sidenote, complexity metrics like MCS, MU/cGy, etc are also independent of BEV flips and rotations and GPR prediction has been successfully implemented relying only on such metrics.

A comparison with some of those other models developed for GPR prediction is presented in Table 3 of Ono et al.^11^ However, a direct comparison is not trivial, since other criteria, other measurement phantoms and other training data has been chosen. Nevertheless, our method allows to drastically reduce the workload posed by PSQA, as only plans below a predicted GPR of 90% need to be measured using a phantom and the LinAc. The model offers a scalable solution for ensuring treatment safety, particularly in time‐sensitive scenarios such as online adaptive radiotherapy, where conventional measurement‐based QA is often not feasible.

Certain nuances must be considered when interpreting our results. The limited measurement volume must be taken into account. Training the model with the full BEV allows it to learn from parts of the treatment plan that are not actually detected by the phantom, potentially reducing accuracy. However, cropping the treatment plans to include only the regions detected by the IMRT phantom during training might mask the impact of the beam model. For example, a cropped 40 cm × 40 cm field would be processed by the model in the same way as a 20 cm × 20 cm field, which does not reflect the actual differences in the output factor. To test this hypothesis, we compared models trained on cropped and uncropped datasets. The cropped model performed slightly worse than the uncropped one, highlighting the importance of the beam model. This finding reinforces our theory that the model indirectly accounts for the beam model, as cropping the plans to the Delta4 measurement volume removes this information. Final proof of this theory is difficult, as the improved performance of the full BEV might be due to input capacity or model complexity, rather than the effect of the beam model. However, further evidence supporting our hypothesis comes from Valdes et al., who identified a metric associated with “the fraction of the plan delivered at the corners” to be one of the most influential predictive GPR metrics—an aspect which is not directly captured by the employed phantom, the Mapcheck 2 (Sun Nuclear Corporation, USA) since its measurement volume is only 32 cm × 26 cm.^12^

The test set data was strictly split at patient level, so the reported test performance is not affected by arc‐level leakage. Nevertheless, arc‐level splitting was applied between training and validation sets, which may have influenced model selection to a minor degree, since arcs from the same treatment plan are inherently correlated through shared patient anatomy. To quantify arc‐to‐arc correlation within the same plan, we computed the Pearson correlation coefficient between paired arcs across all plans (*r* = 0.49 | 78 pairs) and specifically within the clinically relevant subgroup of plans with a GPR < 95% (*r* = 0.15 | 20 pairs). The higher overall correlation is likely driven by the clustering of high‐GPR plans where both arcs naturally perform similarly. In the clinically critical range, where plan failure detection matters most, arc‐to‐arc correlation is weak, supporting the validity of arc‐level splitting for training and validation in our dataset. Nevertheless, this is a methodological limitation, as the model may have benefited from arc‐level correlation during training and validation, potentially leading to a minor overestimation of validation performance.

There are several limitations to the current approach, including the relatively small and unbalanced dataset, the complexity of the model's architecture and the measurement uncertainty for GPR.

The main limitation stems from the complexity of the model and computational resources. Due to GPU constraints, we were unable to train with larger batch sizes, a higher resolution (leaf positions were rounded to 1 mm integers), more data or additional training epochs, which could have potentially led to better performance.

Our research may also be limited by the fact that variations in kernel size were not investigated due to computational constraints. A (3,4, and 4) kernel was applied to all convolutions, with a temporal size of 3 and a spatial size of 4 × 4. However, given the spatial resolution of 5 mm (leaf width at isocenter) × 1 mm, the two directions were not treated equally.

The sensitivity of the GPR measurement to external factors, such as phantom positioning, temperature fluctuations, and daily LinAc conditions, also poses a challenge. This variability complicates the reproducibility of GPR measurements, which could affect consistency of model predictions. While a stringent 1% 2 mm criterion was necessary to ensure the identification of failing plans, this aggressive standard may not fully account for the practical challenges in real‐world measurements.

Future work will still involve improving the model's generalization ability by increasing the dataset size with valid training data and exploring more advanced architectures.

It was possible to add the trained model to the TPS's scripting environment to directly predict the GPR during the treatment planning process and after finishing a treatment plan to directly aid the PSQA process. Possible future applications could be an ongoing association of measured GPR to treatment plans, allowing continuous learning and increased prediction performance. Having the Model in the TPS could possibly avoid plans with a poor GPR already at an early stage in the planning workflow, that is in the optimization process.

Hyperparameter optimization will be part of a follow up project to further streamline this technique and ultimately implement it in clinical practice.

### 4.2 Visual representation using Grad‐CAM

As Grad‐CAM feedback is primarily visual in nature, visual interpretation becomes necessary for evaluating its findings. Our visual interpretation of our results using Grad‐CAM revealed that dynamic variations in treatment parameters played a more critical role in influencing the model's predictions than static factors such as field size and field shape. Specifically, regions with abrupt modulation, meaning substantial leaf motion between two consecutive segments, or irregular motion patterns seem to show stronger activations in the Grad‐CAM visualizations, suggesting that the model prioritizes dynamic complexity over geometric attributes. This insight aligns with clinical intuition, where dynamic beam adjustments and intensity variations contribute significantly to treatment quality and accuracy. Especially VMAT plans for breast irradiation showed a tendency to perform poorer in the GPR evaluation in our clinic in the past. This may be attributed to the fact that during breast VMAT irradiation, the higher tangential dose rate associated with a larger field opening is suddenly diminished when transitioning to the segments, where the beam path is orthogonal to the breast wall. This rapid reduction in field size and dose rate is implemented to protect the lung (and heart in left‐sided treatments). The interaction between these two dynamics likely contributes to the increased uncertainty observed. Employing a second dose calculation algorithm does not reveal the underlying issue that may be causing the plan to fail.

However, it is important to note that this is just our interpretation, as verifying the Grad‐CAM findings is not a trivial task, since RS arcs are restricted in manipulation options. While it is technically possible, certain adjustments, such as splitting VMAT arcs into smaller sections or removing individual segments, cannot be performed.

A longer observation period is required to assess whether adjusting the leaf speed settings in the TPS improves dosimetric plan quality, as this adjustment would reduce leaf travel between consecutive segments—a factor identified by Grad‐CAM as a potential cause of low GPR. A higher GPR without compromising dose coverage compared to the previous period could be achieved.

The findings emphasize the potential of Grad‐CAM in improving treatment plan evaluation by providing an interpretable visualization of key influencing factors. Future work could leverage this approach to refine treatment planning strategies by minimizing high‐impact deviations in dynamic modulation.

## 5 CONCLUSION

In this study, we proposed a novel approach for predicting GPR in VMAT treatment plans using 3D CNNs. By leveraging the discretized BEV and segment‐specific MU data, our model allows for more specific selection and triggering of PSQA. The results show that the model successfully predicts GPR with sufficient accuracy, enabling the introduction of a threshold that can serve as a criterion for actual validation. Considering both the measurement and prediction uncertainties, a prediction threshold of 90% GPR is sufficient in our case to correctly identify all arcs with a measured GPR below 85%, yielding in 0 false pass cases for the cropped and full BEV. This is demonstrated in Figure 4.

Moreover, the generation of heatmaps overlaid on treatment plan segments by implementation of Grad‐CAM allowed a simple visualization of input areas most influential in the model´s decision‐making. This provided valuable insights into the regions of the treatment plan that influence GPR performance, helping medical physicists interpret the results more effectively. This now enables us to identify and adapt potential weaknesses in the TPS settings like in this case we are going to investigate the maximum leaf speed.

In summary, this approach offers a promising alternative to traditional gamma analysis methods, reducing the need for extensive manual measurements, improving the efficiency of PSQA processes in radiotherapy, and deepening our understanding of the factors influencing GPR. However, hyperparameter optimization will be part of a follow up project to further streamline this technique and ultimately implement it in clinical practice.

## CONFLICT OF INTEREST STATEMENT

The authors declare no conflicts of interest.
